# Author response to the letter to the editor regarding “Headaches and lymphocytic pleocytosis after COVID-19 require the exclusion of encephalitis”

**DOI:** 10.1186/s42466-026-00460-0

**Published:** 2026-02-03

**Authors:** Vincent Umathum, Carolin König, Heidrun H. Krämer, Jens Allendörfer, Anne Schänzer

**Affiliations:** 1 Institute of Neuropathology, Justus-Liebig-University, Giessen, Germany; 2 Institute of Pathology and Molecular Pathology, Bundeswehrkrankenhaus Ulm, Germany; 3 Asklepios Neurological Clinic Bad Salzhausen, Nidda, Germany; 4 Department of Neurology, Justus-Liebig-University Giessen, Giessen, Germany

**Authors response to letter to the editor**

Thank you Dr. Finsterer for your letter regarding the study by Umathum et al. [1]. We appreciate your interest in our study and your insightful observations. In the following response, we will address each of your comments, providing clarification and additional context when necessary.

The author stated that an involvement of the central nervous system (CNS) during the person`s lifetime cannot be ruled out. The clinical diagnosis of an encephalitis was not made, as the initial symptoms of headache and dizziness had completely subsided after the correction of significant hyponatraemia (113 mg/dL under thiazide diuretics).

The author highlights that the possibility of Guillain-Barré syndrome (GBS) of the Bickerstaff encephalitis subtype was not excluded. We found no reliable evidence of GBS in the electroneurographic examination, including F-wave recordings and extensive nerve sonography, including the brachial plexus. Following a thorough review of the clinical evidence, there were also no clear indications of Bickerstaff encephalitis. Furthermore, the GQ1b antibodies were negative.

The author mentioned that a cerebral lymphoma was not ruled out. The histological analysis of the post-mortem brain revealed T-lymphocytic infiltrates with perivascular predominance, primarily in the basal ganglia and brain stem in addition to an involvement of the cranial and peripheral nerves. There was no indication of lymphoid cells. In further analysis, T-cell clonality testing revealed no rearrangement of the T-cell receptor beta (TCRB) or gamma (TCRG) genes, indicating a polyclonal T-cell population. Furthermore, Epstein–Barr virus (EBV) in situ hybridisation was negative. In summary our findings are consistent with an immune associated disease.

The author highlights an absence of sufficient description of comorbidities and the course of the disease. Apart from a weight loss of 3 kg over the last few months, there had been no occurrence of B -symptoms. Due to the word restrictions by the journal, we did not describe all pre-existing conditions and medications in detail. The patient, who had multiple underlying conditions, had recovered from the pulmonary symptoms of the previously diagnosed COVID-19 disease. However, based on our clinical assessment and the additional findings reported, we considered steroid therapy to be the most appropriate initial treatment for what was most likely an immune-mediated neuritis associated to SARS-CoV-2 infection. The patient remained stable and was able to walk with minimal assistance throughout, so there was no reason to escalate treatment until her sudden death.

The atrial thrombus was diagnosed by echocardiography, and anticoagulation was performed with low molecular weight heparin.

## Data Availability

Data are available from the corresponding author on reasonable request.
